# Heat Waves and Early Birth: Exploring Vulnerability by Individual‐ and Area‐Level Factors

**DOI:** 10.1029/2025GH001348

**Published:** 2025-04-23

**Authors:** A. Fitch, M. Huang, M. J. Strickland, A. J. Newman, C. Kalb, J. L. Warren, S. Kelley, X. Zheng, H. H. Chang, L. A. Darrow

**Affiliations:** ^1^ Department of Epidemiology, Biostatistics, and Environmental Health School of Public Health University of Nevada, Reno Reno NV USA; ^2^ NSF National Center for Atmospheric Research Boulder CO USA; ^3^ Department of Biostatistics Yale School of Public Health Yale University New Haven CT USA; ^4^ Department of Geography College of Science University of Nevada, Reno Reno NV USA; ^5^ Department of Biostatistics Rollins School of Public Health Emory University Atlanta GA USA

**Keywords:** heat wave, preterm birth, land cover, birth outcomes, early term birth, social deprivation

## Abstract

Extreme heat has been linked to many health outcomes, including preterm and early term birth. We examine associations between acute heat wave exposure and risk of preterm (PTB) (28–36 weeks) or early term (ETB) (37–38 weeks) birth, stratified by individual‐level and area‐level factors. Daily ambient mean temperature was linked to maternal residence in state vital records for preterm and early term births in California, Florida, Georgia, Kansas, Nevada, New Jersey, North Carolina, and Oregon between 1990 and 2017. Heat waves were identified during the four‐day exposure window preceding birth using the 97.5th percentile mean temperature for zip code tabulation areas (ZCTA). We used a time‐stratified case‐crossover design, restricted to the warm season (May through September) and stratified by maternal age, maternal education, ZCTA‐level impervious land cover or social deprivation index. We pooled estimated odds ratios across states using inverse‐variance weighting. The PTB and ETB analyses included up to 945,836 and 2,966,661 cases, respectively. Heat‐related ETB risk was consistently highest among women <25 years of age, women with ≤high school education, and women living in areas of higher social deprivation and impervious land cover. PTB associations were also elevated in these subgroups, but positive associations were also observed among older, more educated mothers, and in areas with less social deprivation. Across all subgroups and outcomes, the change in odds associated with heat waves ranged from no increase to a 7.9% increase. Heat‐related early term birth risk is enhanced among subgroups associated with socioeconomic disadvantage, but patterns of vulnerability were less consistent for preterm birth.

## Key Points

- Heat waves are associated with a modest acute increase in risk of preterm and early term birth (ETB)
- Stronger ETB effects are seen in younger, less educated mothers living in areas with more social deprivation and impervious land cover
- For preterm birth, the association with heat waves is less consistently linked to socioeconomic disadvantage

## 1 Introduction

Early birth is a leading contributor to infant mortality and a variety of health and behavioral effects (Kardatzke et al., 2017; Manuck et al., 2016; Saigal & Doyle, 2008; Shapiro‐Mendoza et al., 2008). While the health consequences of early term birth (ETB) are typically less severe than for preterm birth (PTB), the outcome affects almost three times as many infants in the US (29% vs. 10%) (Osterman et al., 2023), resulting in significant morbidity, both short‐ and long‐term, at the population level (Crump et al., 2013; Martin et al., 2019; Melamed et al., 2009). A growing body of literature demonstrates that acute heat wave exposure in the final days or week of pregnancy is associated with both preterm birth and early term birth (Bekkar et al., 2020; Chersich et al., 2020; Fitch et al., 2025). The magnitude of the association varies across studies and appears to depend on a variety of factors, including study design, heat wave definition, and study population.

Previous research has shown that the acute effect of extreme heat exposure on early birth may vary among different population subgroups based on sociodemographic characteristics such as maternal age, race/ethnicity, income, or education level (Chersich et al., 2020; Huang et al., 2021; Ilango et al., 2020; Son et al., 2022; Sun et al., 2019). Some studies have found that area‐level deprivation, in addition to individual‐level socioeconomic disadvantage, also modifies the association (Cushing et al., 2022; Jiao et al., 2023).

Given that heat waves are increasing in frequency, intensity, and duration (US EPA, 2021), it is important to identify modifiable risk and protective factors, such as residential greening or other heat mitigation practices, that could be targets for intervention. There is reason to believe that differences in land cover, including heat‐trapping or heat‐generating surfaces and structures or green space, could exacerbate or mitigate the effects of extreme heat events (Gunawardena et al., 2017). As a result, it is possible that the association between extreme heat and early birth may also be modified by land cover; however, the evidence for this is somewhat limited and mixed (Cushing et al., 2022; Jiao et al., 2023; Son et al., 2022; Sun et al., 2019, 2020).

In this study, we conducted a time‐stratified case crossover analysis to estimate associations between acute heat wave exposure and early birth in stratified analyses exploring both individual‐ and area‐level modifying factors. We use a novel, high‐resolution meteorological data set and a large, contemporary, multi‐state birth cohort, which includes almost one million preterm births and almost three million early term births across eight states.

## 2 Methods

### 2.1 Data Sources

#### 2.1.1 Health Outcome Data

Up to 28 years of birth data from 1990 to 2017 (unless otherwise noted) were obtained from state vital records for eight states: California, Florida (2004–2017), Georgia (1994–2017), Kansas, Nevada (1991–2017), New Jersey, North Carolina (2002–2015), and Oregon (see Figure S1 in Supporting Information S1 map). These states were chosen in order to achieve some geographic diversity and because obtaining birth data was logistically and financially feasible. Years vary across states due to completeness of maternal residence data in the birth records. Live, singleton births were included if the maternal residence in the birth record could be linked to a ZCTA from the 2010 US Census shapefile (US Census Bureau, 2012) and if covariates used for stratification were available. We examined PTB (28–36 weeks) and ETB (37–38 weeks) as separate outcomes. Exposure assignment (described below) was based on maternal residence in the birth records: in six states the maternal zip code was linked to ZCTA polygons; in New Jersey and North Carolina, the complete street address was provided in the birth record and these were plotted to align with ZCTA polygons to be consistent with exposure assignment in the other six states.

#### 2.1.2 Exposure Data

We obtained 1 km × 1 km gridded estimates of daily maximum and minimum temperatures from the novel High‐resolution Urban Meteorology for Impacts Data set (HUMID) from 1990 to 2017 (A. Newman et al., 2024a). HUMID incorporates an energy balance urban canopy model into a physics based land‐surface modeling system and uses a bias correction process to ensure proper representation of spatial variability in temperatures in urban areas and across urban‐rural divides (detailed methods are summarized in A. J. Newman et al. (2024b)). We represented these estimates as points in R 4.3.2 and, for each day of the study period, we averaged daily minimum and maximum temperatures among all grid points that fell within each ZCTA polygon boundary in each state in our study using 2010 Census ZCTA state shapefiles (US Census Bureau, 2012). We then exported data to SAS 9.4 for remaining analyses and calculated daily mean temperatures for each ZCTA. On average, ZCTAs in our states contained approximately 200 grid points.

We compared shapefiles in ArcGIS Pro and determined that accounting for changes in ZCTA boundaries over time would be unlikely to improve exposure assignment. ZCTA shapefiles are not available prior to 2000, and the 2000 shapefiles are problematic because ZCTAs include large water bodies, national parks and forests (e.g., Death Valley), and other unpopulated areas where temperatures do not accurately reflect values for the residential areas within the same ZCTA. For this reason, we used the 2010 ZCTA boundaries to assign exposures for the entire study period.

To identify heat waves in each ZCTA we first used a relative threshold to identify hot days: the ZCTA‐specific 97.5th percentile mean temperature over the years 1990–2017. Then we identified multi‐day hot periods, assessing (a) the number of consecutive days in a 4‐day window where the mean daily temperature exceeded the threshold, in a definition that emphasizes heat wave duration and (b) the average degrees above the threshold (AAT) over the 4‐day period, in a definition that captures both heat wave intensity and duration. If the AAT was negative, it was set to zero. (These are described as HW2 and HW3, respectively, in our previous work (Darrow et al., 2024; Huang et al., 2021; Richards et al., 2022)).

### 2.2 Study Design

We conducted a time‐stratified case‐crossover design with the day of the PTB or ETB as the event day and three or four referent days within the same month, matched on day of week. The analysis was limited to the warm season, defined as May 1 through September 30. Case‐crossover is a case‐only design where the cases serve as their own controls, naturally controlling for all time‐invariant confounders (Maclure & Mittleman, 2000). Maternal and pregnancy‐related risk factors, such as hypertension or inter‐pregnancy interval, are inherently adjusted for since contrasts are within person. Although this is a case‐only design, we used all births in each state to construct the daily pregnancy risk‐set to adjust for temporal changes in risk as described below.

### 2.3 Analysis Approach

We calculated odds ratios using conditional logistic regression (SAS 9.4) and then pooled state‐specific estimates using inverse‐variance weighting (R 4.3.2) to obtain summary measures (see Equation S1 in Supporting Information S1) (Fleiss, 1993).

Previous research in this area has demonstrated the potential for bias due to within‐window changes in early birth risk due to seasonal trends in conception (Darrow et al., 2009; Huang et al., 2023) as well as within‐window trends in misclassification of gestational age due to digit preferences in last menstrual period reporting (i.e., the 15th of the month) (Frazier, 1959; Waller et al., 2000). To control for this, we adjusted odds ratios for the average probability of birth on each day among ongoing pregnancies at risk of PTB/ETB using an approach we adapted from Vicedo‐Cabrera et al.’s time‐series analysis (2014). For the outcome of PTB, the adjustment variable *W*_*i*_ was calculated as $W_{i}=\sum_{g=28}^{\;36}\left(Z_{ig}{\times W}_{g}\right)/Z_{i}$, where *Z*_*ig*_ is the ZCTA‐level count of fetuses at risk of PTB at gestational week g on calendar day *i*; *Z*_*i*_ is the ZCTA‐level count of all fetuses on day *i*; and *W*_*g*_ is the probability of birth at each gestational week *g*, calculated from all birth records in the state over the study period. The adjustment term for ETB was similarly calculated, but for the relevant 37–38 gestational weeks at risk: $W_{i}=\sum_{g=37}^{\;38}\left(Z_{ig}{\times W}_{g}\right)/Z_{i}$.

We included all preterm and early term births, regardless of whether labor induction was indicated on the birth record because fetal and maternal conditions that motivate medical intervention may have been caused by or exacerbated by the heat wave (e.g., premature rupture of the membranes, placental problems, maternal chronic disease). In previous analyses of these states, we did not see any meaningful difference in the estimates when induced births (approximately 11% of cases) were excluded (Fitch et al., 2025).

### 2.4 Stratification

#### 2.4.1 Maternal Age and Education

We conducted two sets of stratified analyses to explore effect modification based on individual factors obtained in the birth records: maternal age (<25, 25–34, and 35+ years) and maternal educational attainment (less than high school (<HS), high school diploma/General Educational Development Certificate (HS/GED), and more than high school (>HS)). The analysis stratified by educational attainment included seven of the eight states; North Carolina was excluded because these categories of educational attainment were not available in the birth records. We conducted post‐hoc Wald tests separately for age and education to evaluate presence of effect modification for each heat wave definition and outcome (see Equation S2 in Supporting Information S1).

#### 2.4.2 Social Deprivation Index

The first area‐level factor we explored was socioeconomic disadvantage. We used the Social Deprivation Index (SDI) (Butler et al., 2013; Robert Graham Center, n.d.), which is a composite measure based on seven variables available in the US Census American Community Survey: percent of population living below the federal poverty line, percent of population with less than 12 years of education, percent of population ages 16–64 who are unemployed, percent of households in renter‐occupied units, percent of households in crowded housing units, percent of households with no vehicle, and the percent of single parent families with minor dependents. SDI was the preferred measure for our study because it was designed specifically for ZCTA‐level analyses. We extracted the “SDI_score” value from the 2012 SDI table (rgcsdi‐2008–2012‐zcta.csv) for each ZCTA in the eight states. SDI scores range from one to 100, representing centiles. We dichotomized ZCTAs into what we hereafter refer to as “low deprivation” (SDI score < 70) and “high deprivation” (SDI score ≥ 70) categories. These cutoffs were chosen to ensure comparable numbers of cases in each group. We calculated Z‐scores from the pooled estimates and standard errors for each heat wave definition and outcome to evaluate if the differences between strata were significantly different (see Equation S2 in Supporting Information S1).

#### 2.4.3 Land Cover

The second area‐level analysis focused on impervious land cover. We used land cover data from the National Neighborhood Data Archive (National Neighborhood Data Archive (NaNDA), 2020). We determined the proportion of ZCTA area that was classified as medium‐ or high‐intensity urban development (based on impervious land cover) and then categorized into three groups: <25%, 25%–66%, and >66%. These cutoffs were chosen to achieve meaningful variability in impervious land cover while also including at least 20% of cases in each stratum to ensure sufficient power. Further detail is provided in Figure S3 in Supporting Information S1. Maps from three cities included in the analysis are provided as examples of spatial overlap and variability of SDI and land cover categories (see Figure S4 in Supporting Information S1). We conducted a post‐hoc Wald test to evaluate if the differences between land cover strata were significant for each heat wave definition and outcome.

## 3 Results

### 3.1 Descriptive Results

The preterm birth analyses stratified by maternal age, education, SDI, and land cover included between 871,146 and 945,836 cases. The early term birth analyses included between 2,738,119 and 2,966,661 cases. The number of cases varied due to missing data for stratification variables (overwhelmingly due to missing education data in North Carolina). Approximately 50% of the study population for both outcomes came from the California birth records. Table 1 shows the total number and proportion of cases in each demographic and area‐level subgroup. Table S1 in Supporting Information S1 includes the same data broken down by state. Table 1 also shows the aggregate proportion of all births across the eight states that were preterm and early term by subgroup. The proportion of PTBs was higher among younger and older mothers, those with lower education, those in areas with more impervious land cover, and in higher deprivation areas. The ETB proportion was highest among older mothers and was slightly higher in higher deprivation areas, but no notable differences were seen among other subgroups.

**Table 1:** Study Population by Stratum and Outcome; Percent of All Births by Stratum and Outcome

Subgroup	Preterm^a^ cases		Early term^b^ cases		% Preterm (of all births)^c^	% Early term (of all births)^c^
	N	% of total	N	% of total		
Age						
<25	332,171	*35.1*	927,663	*31.3*	*8.7*	*24.1*
25–34	453,049	*47.9*	1,542,178	*52*	*7.4*	*23.7*
35+	160,502	*17*	496,611	*16.7*	*9*	*27.6*
*Total*	945,722		2,966,452			
Education						
<HS	246,179	*28.3*	645,313	*23.6*	*10.8*	*28*
HS/GED	267,605	*30.7*	799,850	*29.2*	*9.7*	*28.7*
>HS	357,362	*41*	1,292,956	*47.2*	*7.9*	*28.1*
*Total*	871,146		2,738,119			
Land cover						
<25%	482,945	*51.1*	1,600,103	*53.9*	*7.7*	*25.3*
25%–66%	249,588	*26.4*	777,521	*26.2*	*8.1*	*24.9*
>66%	213,306	*22.6*	588,921	*19.9*	*9.1*	*24.8*
*Total*	945,839		2,966,545			
Social deprivation index						
Low deprivation: 1–69	423,564	*44.8*	1,490,961	*50.3*	*7.2*	*24.8*
High deprivation: 70–100	522,305	*55.2*	1,475,700	*49.7*	*9.1*	*25.4*
*Total*	945,869		2,966,661			

^a^ Preterm birth = 28–36 weeks' gestation.

^b^ Early term birth = 37–38 weeks' gestation.

^c^ Aggregate proportion of all births in all states (for the study period) that were preterm (28–36 weeks) and early term (37–38 weeks).

Approximately 6.5% of days in the preterm analyses and 6.7% of days in the early term analyses (both limited to May–September) met the most conservative heat wave definition of >2 consecutive days above the threshold. Approximately 3.1% and 3.2% of days in the preterm and early term analyses, respectively, met the >3 consecutive day definition. Just 1.3% and 1.4% of days in the preterm and early term analyses, respectively, met the most extreme definition of all 4 days in the exposure window over the threshold. Approximately 4% of days had a positive value for the continuous heat wave definition of average degrees above the threshold (AAT). Among the days with a positive AAT, the average value was approximately 0.9°C.

### 3.2 Outcomes

**Figure 1:**
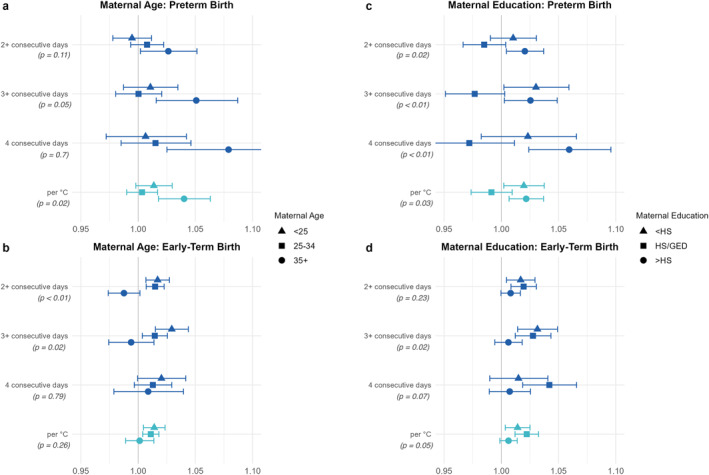
Pooled odds ratios and 95% confidence intervals for the association between heatwaves and early birth. Results stratified by maternal age are shown for preterm birth (a) and early term birth (b). Results stratified by maternal education are shown for preterm birth (c) and early term birth (d). The consecutive‐day heat wave definitions (dark blue) are dichotomous exposure categories. The per °C (teal) definition represents the odds ratio associated with a 1°C increase in the 4‐day average degrees over the 97.5th percentile. Age‐stratified results include all eight states: California, Florida, Georgia, Kansas, Nevada, New Jersey, North Carolina, and Oregon. North Carolina is not included in the education‐stratified results due to lack of data in the birth records. P‐values are presented for tests of heterogeneity across subgroups.

**Figure 2:**
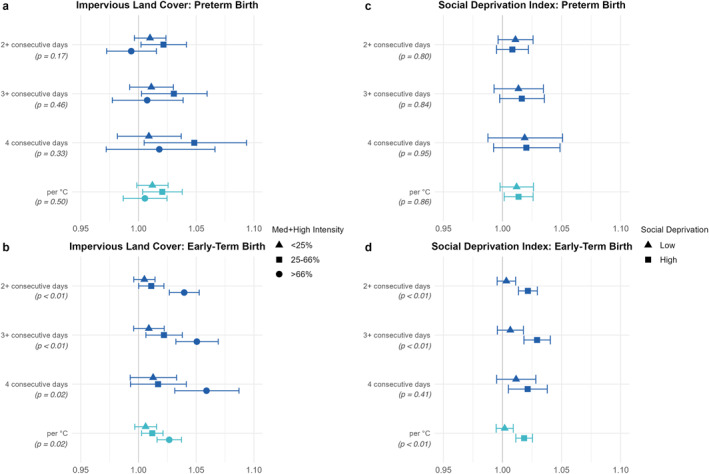
Pooled odds ratios and 95% confidence intervals for the association between heatwaves and early birth. Results stratified by land cover are shown for preterm birth (a) and early term birth (b). Results stratified by social deprivation index are shown for preterm birth (c) and early term birth (d). The consecutive‐day heat wave definitions (dark blue) are dichotomous exposure categories. The per °C (teal) definition represents the odds ratio associated with a 1°C increase in the 4‐day average degrees over the 97.5th percentile. All results include all eight states: California, Florida, Georgia, Kansas, Nevada, New Jersey, North Carolina, and Oregon. P‐values are presented for tests of heterogeneity across subgroups.

With only a few exceptions, the pattern in results was highly consistent across heat wave definitions. For the sake of clarity and consistency, we summarize the stratified results using a single heat wave definition: the odds ratio associated with a one‐degree (Celsius) increase in the 4‐day average mean temperature above the area‐specific 97.5th percentile threshold mean temperature (AAT). Results for all four heat wave definitions are shown in Figures 1 and 2, and full numerical results are presented in Table S2 in Supporting Information S1. State‐specific results for the continuous AAT heat wave definition are presented in Figure S5 in Supporting Information S1 to show the variability and precision of estimates across states. State‐specific numerical results are presented in Tables S3–S6 in Supporting Information S1. Overall Because ETB included three times the number of cases as PTB, estimates were notably more precise for the ETB outcome in all analyses.

#### 3.2.1 Maternal Age

Pooled adjusted odds ratios showed positive associations for heat waves and PTB in the oldest maternal age group (35+ years), OR = 1.040 (1.018, 1.063). The result for the youngest maternal age group (<25 years) was also elevated to a lesser extent, OR = 1.014 (0.998, 1.030). The associations for ETB, however, were elevated for the younger and middle groups of maternal age (OR = 1.014 (1.005, 1.024) and 1.011 (1.004, 1.018), respectively), but not the oldest group. See Figures 1a and 1b. The Wald test for heterogeneity of estimates was significant for PTB (*p* = 0.02), but not ETB (*p* = 0.26).

#### 3.2.2 Maternal Education

The maternal education results consistently showed that those with less education had elevated ORs; this was seen for both PTB and ETB, OR = 1.020 (1.002, 1.037) and OR = 1.014 (1.003, 1.025), respectively. A significant positive association was also observed for PTB among the highest educational attainment group, OR = 1.022 (1.007, 1.037). All results for ETB were in the positive direction, with less difference between the subgroups than was evident for PTB. The ORs for ETB ranged from 1.006 for the highest education group (>HS), to 1.022 for the middle group (HS/GED). See Figures 1c and 1d. The Wald test for heterogeneity of estimates was significant for PTB (*P* = 0.03), and approached significance for ETB (*p* = 0.05).

#### 3.2.3 Land Cover

For PTB, associations with heat waves were highest in the middle category of impervious land cover, OR = 1.021 (1.004, 1.038), although confidence intervals largely overlapped between land cover groups. Results for ETB followed a different pattern where there was evidence of an increasing association with heat waves as impervious land cover increased. We saw no association within the lowest impervious land cover category (OR = 1.006 (0.997, 1.016)), a modest positive association for the middle category (OR = 1.012 (1.003, 1.021)), and a stronger association for the highest category (OR = 1.027 (1.016, 1.037)). See Figures 2a and 2b. We note, however, that the sample size for the highest category of impervious land cover was very small in five of the states, accounting for less than 1% of the data in Florida, Georgia, Kansas, and North Carolina, and less than 5% of the data in Oregon. Wald test results indicated presence of effect modification for ETB (*p* = 0.02), but not PTB (*p* = 0.50).

#### 3.2.4 Social Deprivation Index

For PTB, associations with acute heat wave exposure were similar across the two categories of SDI. ORs for low and high SDI were 1.012 (0.998, 1.026) and 1.014 (1.001, 1.026), respectively. However, for ETB, there was a significant positive association in the higher deprivation group, OR = 1.018 (1.011, 1.025), but not the lower deprivation group, OR = 1.002 (0.995, 1.009). See Figures 2c and 2d. The difference in heat wave odds ratios between the high and low SDI groups was significant for ETB (*p* < 0.01), but not PTB (*p* = 0.86).

## 4 Discussion

This study examined heat waves over three decades in eight geographically diverse states whose populations account for almost one‐third of the U.S. population (US Census Bureau, 2024). Our analyses of preterm and early term birth included almost one‐ and three‐million cases, respectively, and utilized high‐resolution meteorology data linked to maternal residence by ZCTA. The results showed modestly elevated associations between heat waves and early birth and suggest that those associations are stronger among socioeconomically disadvantaged populations. For PTB, the highest heat‐related risk was less consistently linked to socioeconomic disadvantage, as associations were also evident among those living in low deprivation areas, older mothers, and more educated mothers.

For the association of acute heat wave exposure and ETB, we found evidence of effect modification across all stratification variables. Stronger effects were evident among younger and less educated mothers as well as those living in areas of higher social deprivation and higher impervious land cover. These ETB trends were consistent with our expectations; we hypothesized that the effect would be stronger for those with less education or those in higher deprivation areas, because they may be less likely to have reliable central air conditioning (AC) or may utilize it more sparingly to save on utility costs during the hottest days of the year. They may also be less likely to own a vehicle, resulting in more outdoor exposure to heat during active transportation. In addition, higher impervious land cover generally occurs in dense urban environments where we are more likely to see pockets of inner‐city poverty as well as urban heat islands, both of which might magnify the association.

Our results for PTB are somewhat different, and the trends are less clear. In general, the preterm results are less precise due to lower outcome counts. Similar to ETB, we observed elevated associations for younger mothers and those with the least education. In contrast, however, we also found elevated associations for older mothers and those with the most education. Our finding of elevated heat wave‐PTB risk in older mothers is consistent with at least four other studies from the US and other countries (Cox et al., 2016; Min et al., 2024; Son et al., 2022; Wang et al., 2013). Pregnancies in older mothers are generally considered higher risk due to increased rates of a variety of maternal and neonatal complications (Hochler et al., 2023), some of which could be exacerbated by a heat wave. A stronger heat‐early birth association in younger mothers is more frequently reported (Chersich et al., 2020; Son et al., 2022), though there are a few studies that found no difference by maternal age (Huang et al., 2021; Ilango et al., 2020). We found few studies exploring effect modification by maternal age for ETB (Spolter et al., 2020).

Although it is well documented that PTB is more common among mothers with less education, whether or not education serves as an effect modifier for an acute effect of heat waves is less clear. Some studies report no difference by maternal education (Basu et al., 2010; Huang et al., 2021) while others report relatively stronger effects in less educated populations (Schifano et al., 2013; Y. Sun et al., 2020). These differences may result at least in part from differing cutoffs for education levels. Also, education is correlated with other factors, such as age. Our finding of a stronger heat‐PTB effect in the highest education stratum appears unique in the literature.

Consistent evidence is emerging to show that land cover is associated with PTB risk. For example, a Canadian study found that increased greenness was associated with a decreased likelihood of PTB (among other birth outcomes) and that the association was still evident even after adjustment for correlated built environment factors such as noise, neighborhood walkability, distance to nearest park, and air pollution (Hystad et al., 2014). Though it is a relatively newer area of research, several studies have reported that land cover may modify the heat‐early birth association, with stronger effects in less green areas, though effects may differ in urban and rural areas (Min et al., 2024; Son et al., 2022; Sun et al., 2020). In our study, while those in the highest impervious land cover category had the strongest association for ETB, such a trend was not evident for PTB, which showed more similar effect estimates between high and low impervious land cover categories.

In a recent study of the association between heat waves and premature rupture of membranes (PROM), Jiao et al. (2023) found that green space and census‐tract‐level AC use (based on smart meter data) appeared to mitigate the adverse effects of heat on PROM risk. Lack of fine‐scale air conditioning data in our states prevented us from exploring it directly as an effect modifier for PTB/ETB; however, a recent study of intra‐urban variation of air conditioning access in the US reported that AC access was disproportionately lower in more socially vulnerable census tracts and in areas with stronger urban heat island effects (Romitti et al., 2022). While we found evidence of stronger associations among those in higher social deprivation areas in the ETB results, we found no difference between groups for PTB, with both low and high social deprivation subgroups showing similar positive associations. Our PTB results differ from findings in several previous studies (in Rome, North Carolina, and Houston, Texas), which report stronger heat‐preterm birth effects in areas with more, but not less, socioeconomically disadvantage (Asta et al., 2019; Cushing et al., 2022; Son et al., 2022). Differences across subgroups in our study and differences between our study results and previous research could also be partially due to differing levels of air pollution. Air pollution may mediate the association between heat waves and early birth, as extreme heat can exacerbate air pollution, which may, in turn, increase early birth risk (Bekkar et al., 2020).

We acknowledge several limitations to our research related to the use of ZCTAs to assign exposure data. While, the HUMID data set provides high‐resolution temperature estimates, we calculated averages at the ZCTA level to align with residence information provided in the birth records. Such aggregation reduces the precision of the exposure measurement. Some ZCTAs, particularly in urban areas, are geographically quite small, resulting in temperature averages that are reliant on just a few gridpoint estimates. We note, however, that we assigned heat wave exposures based on a relative threshold; even with some inaccuracies in the ZCTA boundaries or temperature averages, the days identified as being over the 97.5th percentile were likely to have been the hottest days.

There is also error in the outcome measurement as we used gestational ages from birth records. Methods of determining gestational age have changed from 1990, when they were primarily determined by maternal reporting of onset date of the last menstrual period, to 2017, when they are primarily based on ultrasound measurements. These issues are mitigated somewhat by our focus on acute effects and restriction of analysis to within‐month exposure contrasts; these outcome errors are unlikely to drive spurious associations due to their independence from short‐term exposure variation.

Future research in this area should explore whether or not access to and utilization of air conditioning may explain some of the associations observed. Heat‐related illness risk is higher among those without air conditioning (Cardoza et al., 2020), therefore air conditioning may also modify the association between extreme heat and birth outcomes. However, air conditioning data are not available at a fine spatial scale outside of certain cities; the American Community Survey has not asked about household cooling since the 1980s. Such data sources need to be developed.

The main strengths of our study include the large study population, owing to both the number of states and the long study period, the examination of both PTB and ETB outcomes, and the novel high‐resolution exposure data set that better captures variability in temperatures in urban areas and across urban‐rural interfaces. The results of the stratified analyses for ETB were consistent with our hypothesis that the effect would be stronger in populations and areas with more socioeconomic disadvantage and in areas with more heat‐trapping land cover. For PTB, socioeconomic factors were less consistently linked to stronger heat‐PTB results as we also observed elevated associations in older women, more educated women, and women in areas with lower socioeconomic deprivation. Our PTB results strengthen the evidence for increased heat‐related PTB risk in mothers over age 35.

Our findings may have implications for prenatal management or community‐level adaptive capacity efforts in socioeconomically disadvantaged areas and urban centers. While the influence of air conditioning access on the heat‐early birth association has yet to be researched in a large, multi‐state study like ours, previous research has shown that both central air conditioning and window air conditioning can reduce the risk of heat exhaustion (Cardoza et al., 2020). Health care providers can recommend pregnant people invest in window units if their homes do not have central air conditioning. In heat wave‐prone areas in the US, efforts can be made to ensure patients know if they can secure assistance for purchase of window/wall cooling units through the Low Income Home Energy Assistance Program benefits (US Department of Health and Human Services, n.d.). In addition, with only a few exceptions, landlords in the US do not have a legal obligation to provide air conditioning under “habitable condition” regulations (Natural Resources Defense Council, 2022). A few examples of such requirements do exist and could be models for other states. Finally, there is evidence that low‐income residents are less likely to be aware of heat warnings (Madrigano et al., 2015), highlighting the need for improved crisis and emergency risk communication for vulnerable populations.

## Conflict of Interest

The authors declare no conflicts of interest relevant to this study.

## Supporting information

Supporting Information S1

## Data Availability

The meterology data used to determine heat wave exposure in the study are publicly available in The High‐resolution Urban Meteorology for Impacts Data set (HUMID), developed by the National Center for Atmostpheric Research (A. Newman et al., 2024a). Birth records were obtained through data use agreements with state health departments in California, Florida, Georgia, Kansas, Nevada, New Jersey, North Carolina, and Oregon. The agreements prevent sharing birth records due to patient confidentiality concerns. Odds ratios and 95% confidence intervals for pooled results and R code for plotting the results are available in this Zenodo repository: https://doi.org/10.5281/zenodo.15002786 (Richards & Fitch, 2025).
