# Supplementary material for: Heat Waves and Early Birth: Exploring Vulnerability by Individual‐ and Area‐Level Factors
Source: Geohealth. 2025 Apr 23;9(4):e2025GH001348. doi: 10.1029/2025GH001348 (PMC12015208; doi:10.1029/2025GH001348)
Supplement: Supplementary file 1 — Supporting Information S1 [file GH2-9-e2025GH001348-s001.pdf]

*Geo Health*

Supporting Information for

# Heat Waves and Early Birth: Exploring Vulnerability by Individual- and Area-level Factors

A. Fitch[1], M. Huang[1], M.J. Strickland[1], A.J. Newman[2], C. Kalb[2], J.L. Warren[3], S. Kelley[4], X. Zheng[5], H.H. Chang[5], and L.A. Darrow[1]

[1]Department of Epidemiology, Biostatistics, and Environmental Health, School of Public Health, University of Nevada, Reno. [2]NSF National Center for Atmospheric Research. [3]Department of Biostatistics, Yale School of Public Health, Yale University. [4]Department of Geography, College of Science, University of Nevada, Reno. [5]Department of Biostatistics, Rollins School of Public Health, Emory University.

## Contents of this file


| | |
|---|---|
| Figure S1: Map of US showing eight states included in the analysis | 2 |
| Equation S1: Inverse-variance weighting | 3 |
| Equation S2: Tests for heterogeneity | 3 |
| Figure S3: Visual description of how land cover was categorized in our study | 4 |
| Figure S4: Sample maps: Land cover and social deprivation | 5 |
| Figure S5: State-specific results plot | 6 |
| Table S1: State-specific study population (number and percent of cases) by stratum | 7-8 |
| Table S2: Pooled results for all stratification categories | 9 |
| Table S3: State-specific results stratified by maternal age | 10-11 |
| Table S4: State-specific results stratified by maternal education | 12 |
| Table S5: State-specific results stratified by land cover | 13-14 |
| Table S6: State-specific results stratified by social deprivation index | 15 |


## Introduction

In this document, we provide supplemental text, figures, and tables for the main manuscript, including tables of numerical result for plots included in the manuscript.

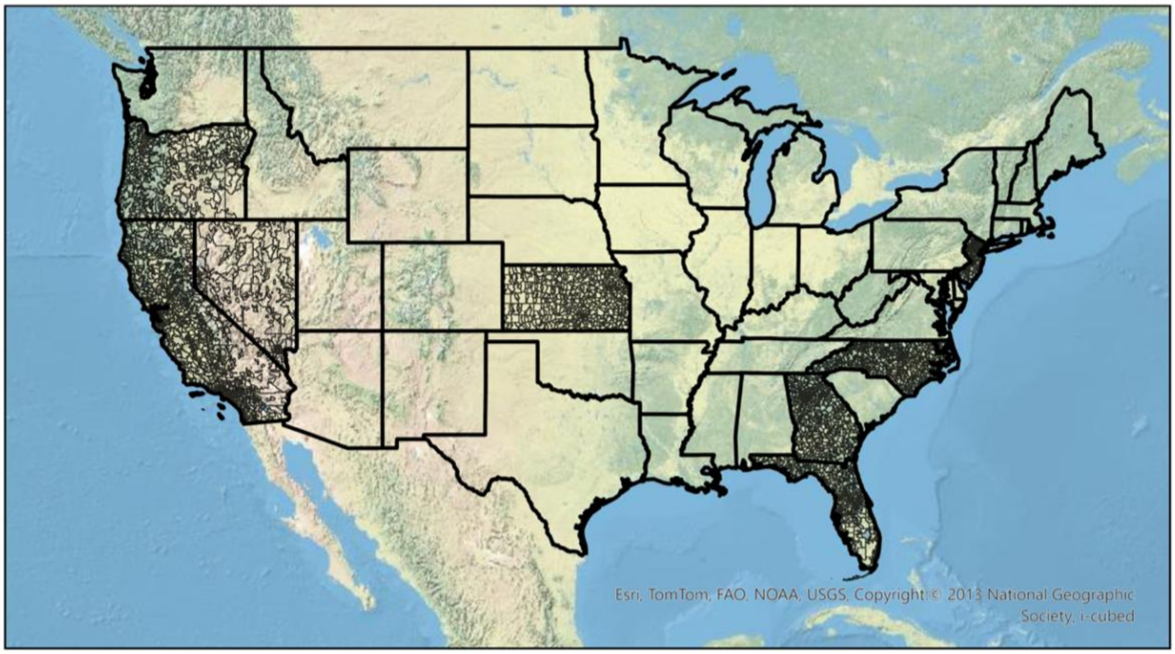


**Figure S1:** Eight states included in the analysis. California, Oregon, and Nevada (west). Kansas (central). Florida, Georgia, and North Carolina (southeast). New Jersey (northeast).

**Equation S1: Inverse-variance weighting** (Fleiss, 1993)

Pooled estimate

$$\bar{Y} = \frac{\Sigma W_c Y_c}{\Sigma W_c}$$

Standard error

$$\mathrm{SE}(\bar{Y}) = (\Sigma W_c)^{-1/2}$$

Where *C* is the number of studies (states), *Y* is the effect measure, and *W* is the reciprocal of its variance.

**Equation S2: Heterogeneity tests**

Wald test: Hypothesis test of interest:

$$H_0: \mathrm{C}\widehat{\boldsymbol{\beta}} = \mathbf{0}$$
$$H_1: \mathrm{C}\widehat{\boldsymbol{\beta}} \neq \mathbf{0}$$

Assumption of asymptotic multivariate normality of the estimator:

$$\widehat{\boldsymbol{\beta}} \approx \mathrm{MVN}(\boldsymbol{\beta}, \Sigma)$$

We will be approximating this because we only have the diagonal of this matrix (i.e., the standard errors)

Contrast matrix needed to test if $\beta_1 = \beta_2 = \beta_3$:

$$\mathrm{C} = \begin{pmatrix} 1 & -1 & 0 \\ 0 & 1 & -1 \end{pmatrix}$$

Wald test statistic:

$$W = \left(\mathrm{C}\widehat{\boldsymbol{\beta}} - 0\right)^{\mathrm{T}} \left(\mathrm{C}\Sigma\mathrm{C}^{\mathrm{T}}\right)^{-1} \left(\mathrm{C}\widehat{\boldsymbol{\beta}} - 0\right)$$

Under the null distribution, $W \approx \chi_2^2$, allowing us to calculate a p-value

For SDI, which only had 2 subgroups (high and low), a Z score was used to test the null hypothesis $\beta_1 = \beta_2$:

$$Z = \frac{\beta_1 - \beta_2}{\sqrt{(SE\beta_1)^2 + (SE\beta_2)^2}}$$

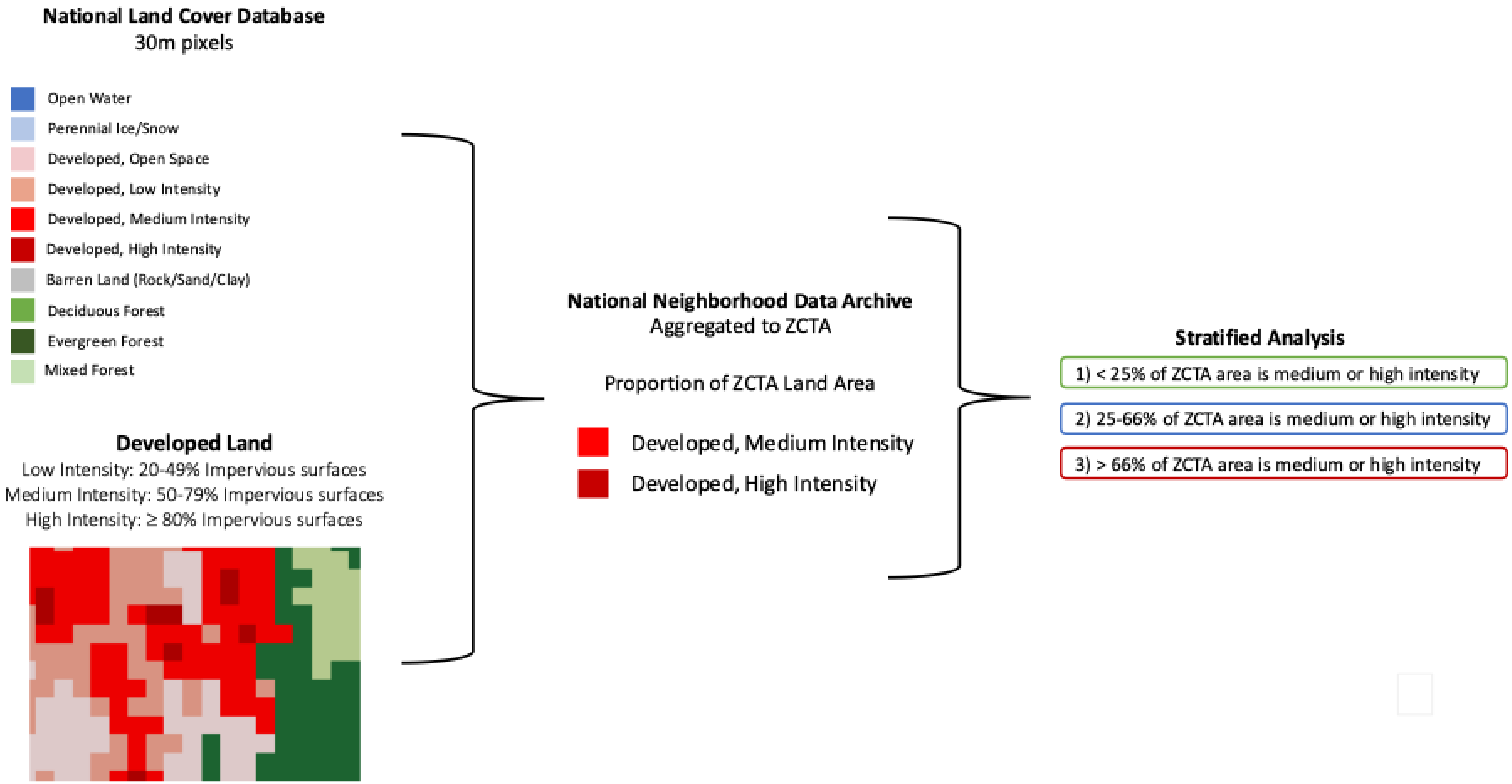


**Figure S3.** Graphic description of how we categorized land cover based on The National Land Cover Database (NLCD) and the National Neighborhood Data Archive (NaNDA). NLCD categorizes 30-meter pixels by land cover. NaNDA aggregates to the ZCTA level and assigns percentage of land area in the ZCTA that belongs in each NLCD category. We summed the NLCD categories of 'Developed, Medium Intensity' (50-79% of area is impervious) and 'Developed, High Intensity' (≥80% of area is impervious) and then split **i**nto three categories. Our categories can be interpreted as the proportion of ZCTA area that is ≥50% impervious land cover.

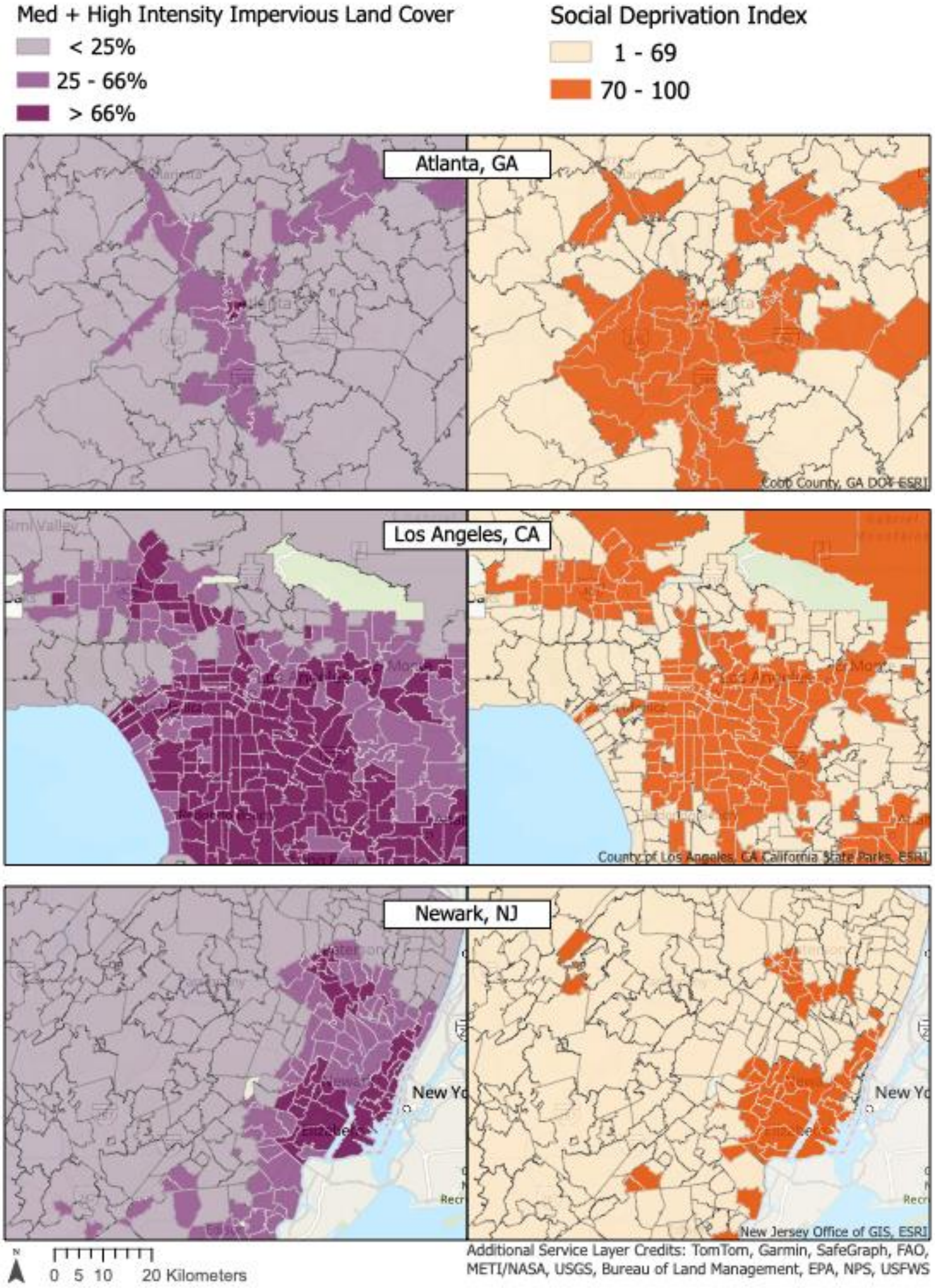


**Figure S4.** Maps for Atlanta, Los Angeles, and Newark showing examples of the geographic distribution of land cover and SDI categories.

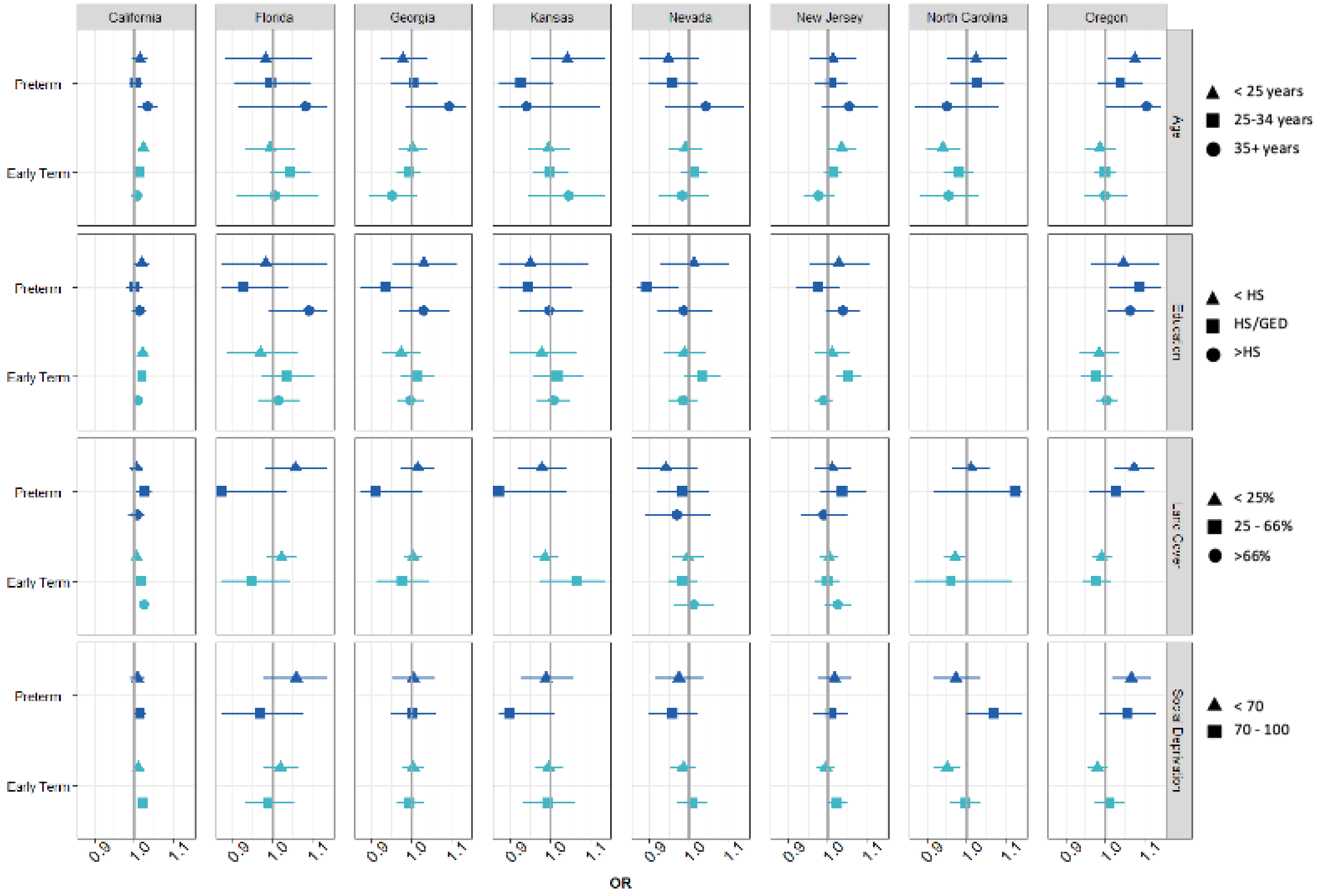


**Figure S5.** State-specific results odds ratios for 1°C increase in 4-day average temperature above the threshold. North Carolina was not included in the maternal education analyses because these categories of educational attainment were not in the birth record. Results not presented for highest stratum of land cover for Florida, Georgia, Kansas, North Carolina, and Oregon due to low sample size (<5% of the data)

Table S1: Number and percent of cases by state and stratum

| Preterm Cases | California | | Florida | | Georgia | | Kansas | | Nevada | |
|---|---|---|---|---|---|---|---|---|---|---|
| **Age** | N | % | N | % | N | % | N | % | N | % |
| <25 | 181,255 | *35.1* | 32,428 | *33.0* | 42,664 | *40.23* | 11,889 | *40.81* | 10,393 | *35.63* |
| 25-34 | 244,639 | *47.4* | 48,672 | *49.5* | 48,486 | *45.7* | 13,851 | *47.5* | 13,957 | *47.9* |
| 35+ | 90,595 | *17.5* | 17,235 | *17.5* | 14,912 | *14.1* | 3,395 | *11.7* | 4,817 | *16.5* |
| *Total* | *516,489* | | *98,335* | | *106,062* | | *29,135* | | *29,167* | |
| **Education*** | | | | | | | | | | |
| <HS | 168,675 | *33.5* | 18,423 | *18.9* | 24,651 | *24.0* | 5,775 | *19.9* | 8,014 | *28.4* |
| HS/GED | 146,795 | *29.2* | 33,178 | *34.0* | 34,448 | *33.5* | 8,861 | *30.5* | 9,492 | *33.6* |
| >HS | 187,913 | *37.3* | 45,997 | *47.1* | 43,726 | *42.5* | 14,375 | *49.6* | 10,715 | *38.0* |
| *Total* | *503,383* | | *97,598* | | *102,825* | | *29,011* | | *28,221* | |
| **Land Cover** | | | | | | | | | | |
| <25% | 173,818 | *33.6* | 67,370 | *68.5* | 95,510 | *90.1* | 25,558 | 87.8 | 8,107 | *27.8* |
| 25%-66% | 162,354 | *31.4* | 30,098 | *30.6* | 10,291 | *9.7* | 3,473 | 11.9 | 11,191 | *38.3* |
| >66% | 180,410 | *34.9* | 864 | *0.9* | 252 | *0.2* | 90 | 0.3 | 9,914 | *33.9* |
| *Total* | *516,582* | | *98,332* | | *106,053* | | *29,121* | | *29,212* | |
| **Social Deprivation Index** | | | | | | | | | | |
| Low Deprivation: 1-69 | 181,014 | *35.0* | 55,958 | *56.9* | 55,522 | *52.3* | 21,827 | 74.9 | 13,058 | *44.7* |
| High Deprivation: 70-100 | 335,568 | *65.0* | 42,381 | *43.1* | 50,540 | *47.7* | 7,308 | 25.1 | 16,154 | *55.3* |
| *Total* | *516,582* | | *98,339* | | *106,062* | | *29,135* | | *29,212* | |
| **Early Term Cases** | **California** | | **Florida** | | **Georgia** | | **Kansas** | | **Nevada** | |
| **Age** | N | % | N | % | N | % | N | % | N | % |
| <25 | 456,411 | *30.5* | 112,091 | *30.9* | 127,548 | *37.2* | 38382 | *37.1* | 32,792 | *34.4* |
| 25-34 | 775,278 | *51.8* | 189,677 | *52.3* | 170,332 | *49.6* | 53563 | *51.8* | 48,795 | *51.2* |
| 35+ | 266,195 | *17.8* | 61,214 | *16.9* | 45,350 | *13.2* | 11540 | *11.2* | 13,723 | *14.4* |
| *Total* | *1,497,884* | | *362,982* | | *343,230* | | *103,485* | | *95,310* | |
| **Education*** | | | | | | | | | | |
| <HS | 416,092 | *28.5* | 58,302 | *16.2* | 70,536 | *21.1* | 17819 | *17.3* | 23,235 | *25.0* |
| HS | 405,248 | *27.7* | 114,298 | *31.7* | 106,385 | *31.9* | 29100 | *28.2* | 30,648 | *33.0* |
| >HS | 640,838 | *43.8* | 188,230 | *52.2* | 156,981 | *47.0* | 56168 | *54.5* | 39,081 | *42.0* |
| *Total* | *1,462,178* | | *360,830* | | *333,902* | | *103,087* | | *92,964* | |
| **Land Cover** | | | | | | | | | | |
| <25% | 519,451 | *34.7* | 253,281 | *69.8* | 312,646 | *91.1* | 91,843 | *88.8* | 29,550 | *31.0* |
| 25%-66% | 488,641 | *32.6* | 106,287 | *29.3* | 29,822 | *8.7* | 11,286 | *10.9* | 36,014 | *37.8* |
| >66% | 489,946 | *32.7* | 3,405 | *0.9* | 723 | *0.2* | 293 | *0.3* | 29,786 | *31.2* |
| *Total* | *1,498,038* | | *362,973* | | *343,191* | | *103,422* | | *95,350* | |
| **Social Deprivation Index** | | | | | | | | | | |
| Low Deprivation: 1-69 | 596,703 | *39.8* | 221,437 | *61.0* | 193,918 | *56.5* | 80,367 | *77.7* | 47,801 | *50.1* |
| High Deprivation: 70-100 | 901,335 | *60.2* | 141,550 | *39.0* | 149,312 | *43.5* | 23,118 | *22.3* | 47,549 | *49.9* |
| *Total* | *1,498,038* | | *362,987* | | *343,230* | | *103,485* | | *95,350* | |

Table S1: Number and percent of cases by state and stratum

| **Preterm Cases** | **New Jersey** | | **North Carolina** | | **Oregon** | | **TOTAL** | |
|---|---|---|---|---|---|---|---|---|
| **Age** | N | % | N | % | N | % | N | % |
| <25 | 21,432 | *26.28* | 21492 | *38.43* | 10,618 | *36.53* | 332,171 | *35.1* |
| 25-34 | 43,033 | *52.8* | 26333 | *47.1* | 14,078 | *48.4* | 453,049 | *47.9* |
| 35+ | 17,078 | *20.9* | 8097 | *14.5* | 4,373 | *15.0* | 160,502 | *17.0* |
| *Total* | *81,543* | | *55,922* | | *29,069* | | *945,722* | |
| **Education*** | | | | | | | | |
| <HS | 14,239 | *17.5* | - | - | 6,402 | *22.4* | 246,179 | *28.3* |
| HS/GED | 25,826 | *31.7* | - | - | 9,005 | *31.5* | 267,605 | *30.7* |
| >HS | 41,423 | *50.8* | - | - | 13,213 | *46.2* | 357,362 | *41.0* |
| *Total* | *81,488* | | | | *28,620* | | *871,146* | |
| **Land Cover** | | | | | | | | |
| <25% | 39,366 | *48.3* | 53,927 | *96.4* | 19,289 | *66.3* | 482,945 | *51.1* |
| 25%-66% | 21,593 | *26.5* | 1,960 | *3.5* | 8,628 | *29.7* | 249,588 | *26.4* |
| >66% | 20,584 | *25.2* | 35 | *0.1* | 1,157 | *4.0* | 213,306 | *22.6* |
| *Total* | *81,543* | | *55,922* | | *29,074* | | *945,839* | |
| **Social Deprivation Index** | | | | | | | | |
| Low Deprivation: 1-69 | 43,931 | *53.9* | 31,939 | *57.1* | 20,315 | *69.9* | 423,564 | *44.8* |
| High Deprivation: 70-100 | 37,612 | *46.1* | 23,983 | *42.9* | 8,759 | *30.1* | 522,305 | *55.2* |
| *Total* | *81,543* | | *55,922* | | *29,074* | | *945,869* | |
| **Early Term Cases** | **New Jersey** | | **North Carolina** | | **Oregon** | | **TOTAL** | |
| **Age** | N | % | N | % | N | % | N | % |
| <25 | 61,826 | *22.2* | 62,906 | *35.6* | 35,707 | *33.1* | 927,663 | *31.3* |
| 25-34 | 157,866 | *56.6* | 90,039 | *50.9* | 56,628 | *52.4* | 1,542,178 | *52.0* |
| 35+ | 59,038 | *21.2* | 23,905 | *13.5* | 15,646 | *14.5* | 496,611 | *16.7* |
| *Total* | *278,730* | | *176,850* | | *107,981* | | *2,966,452* | |
| **Education*** | | | | | | | | |
| <HS | 38,440 | *13.8* | - | - | 20,889 | *19.6* | 645,313 | *23.6* |
| HS | 81,697 | *29.3* | - | - | 32,474 | *30.5* | 799,850 | *29.2* |
| >HS | 158,399 | *56.9* | - | - | 53,259 | *50.0* | 1,292,956 | *47.2* |
| *Total* | *278,536* | | | | *106,622* | | *2,738,119* | |
| **Land Cover** | | | | | | | | |
| <25% | 148,575 | *53.3* | 171,659 | *97.1* | 73,098 | *67.7* | 1,600,103 | *53.9* |
| 25%-66% | 69,520 | *24.9* | 5,096 | *2.9* | 30,855 | *28.6* | 777,521 | *26.2* |
| >66% | 60,635 | *21.8* | 95 | *0.1* | 4,038 | *3.7* | 588,921 | *19.9* |
| *Total* | *278,730* | | *176,850* | | *107,991* | | *2,966,545* | |
| **Social Deprivation Index** | | | | | | | | |
| Low Deprivation: 1-69 | 168,157 | *60.3* | 105,963 | *59.9* | 76,615 | *70.9* | 1,490,961 | *50.3* |
| High Deprivation: 70-100 | 110,573 | *39.7* | 70,887 | *40.1* | 31,376 | *29.1* | 1,475,700 | *49.7* |
| *Total* | *278,730* | | *176,850* | | *107,991* | | *2,966,661* | |

Table S1: Study population by state. For each state, we present the number and percent of cases that fall within each stratum. Numbers may vary across stratification categories within the state due to data availability for that variable. The total column includes all eight states.

*These categories of educational achievement data not available in North Carolina birth records.

Table S2: Pooled results for all outcomes, strata, and heat wave definitions.

| Outcome | Category | Level | 2+ consecutive days | 3+ consecutive days | 4 consecutive days | per °C |
|---|---|---|---|---|---|---|
| Preterm | Age | <25 | 0.995 (0.978,1.012) | 1.011 (0.987,1.035) | 1.007 (0.972,1.042) | 1.014 (0.998,1.030) |
| Preterm | Age | 25-34 | 1.008 (0.994,1.022) | 1.000 (0.980,1.021) | 1.015 (0.985,1.046) | 1.003 (0.990,1.017) |
| Preterm | Age | 35+ | 1.026 (1.002,1.051) | 1.051 (1.016,1.087) | 1.079 (1.025,1.136) | 1.040 (1.018,1.063) |
| Preterm | Education | <HS | 1.010 (0.990,1.031) | 1.030 (1.002,1.059) | 1.023 (0.982,1.066) | 1.020 (1.002,1.037) |
| Preterm | Education | HS/GED | 0.985 (0.967,1.004) | 0.977 (0.951,1.003) | 0.972 (0.934,1.011) | 0.991 (0.974,1.009) |
| Preterm | Education | >HS | 1.021 (1.004,1.037) | 1.025 (1.003,1.049) | 1.059 (1.024,1.096) | 1.022 (1.007,1.037) |
| Preterm | SDI | Low | 1.011 (0.996,1.026) | 1.014 (0.993,1.035) | 1.019 (0.988,1.051) | 1.012 (0.998,1.026) |
| Preterm | SDI | High | 1.008 (0.995,1.022) | 1.016 (0.998,1.035) | 1.020 (0.992,1.049) | 1.014 (1.001,1.026) |
| Preterm | Land Cover | <25% | 1.010 (0.996,1.024) | 1.011 (0.992,1.030) | 1.009 (0.982,1.037) | 1.012 (0.999,1.026) |
| Preterm | Land Cover | 25-66% | 1.022 (1.002,1.041) | 1.031 (1.003,1.059) | 1.048 (1.005,1.094) | 1.021 (1.004,1.038) |
| Preterm | Land Cover | >66% | 0.994 (0.972,1.015) | 1.008 (0.977,1.039) | 1.018 (0.972,1.066) | 1.006 (0.987,1.025) |
| Early Term | Age | <25 | 1.017 (1.007,1.027) | 1.029 (1.015,1.044) | 1.020 (1.000,1.042) | 1.014 (1.005,1.024) |
| Early Term | Age | 25-34 | 1.015 (1.007,1.023) | 1.015 (1.004,1.026) | 1.013 (0.997,1.029) | 1.011 (1.004,1.018) |
| Early Term | Age | 35+ | 0.988 (0.974,1.002) | 0.994 (0.974,1.014) | 1.009 (0.979,1.040) | 1.001 (0.989,1.014) |
| Early Term | Education | <HS | 1.017 (1.005,1.029) | 1.032 (1.014,1.049) | 1.015 (0.990,1.041) | 1.014 (1.003,1.025) |
| Early Term | Education | HS/GED | 1.019 (1.008,1.031) | 1.028 (1.012,1.043) | 1.042 (1.019,1.066) | 1.022 (1.012,1.033) |
| Early Term | Education | >HS | 1.008 (1.000,1.017) | 1.006 (0.994,1.018) | 1.007 (0.989,1.025) | 1.006 (0.999,1.014) |
| Early Term | SDI | Low | 1.003 (0.995,1.011) | 1.007 (0.996,1.018) | 1.012 (0.995,1.028) | 1.002 (0.995,1.009) |
| Early Term | SDI | High | 1.021 (1.014,1.029) | 1.029 (1.018,1.040) | 1.021 (1.005,1.038) | 1.018 (1.011,1.025) |
| Early Term | Land Cover | <25% | 1.005 (0.996,1.014) | 1.009 (0.996,1.022) | 1.013 (0.993,1.033) | 1.006 (0.997,1.016) |
| Early Term | Land Cover | 25-66% | 1.011 (1.000,1.022) | 1.022 (1.006,1.038) | 1.017 (0.993,1.041) | 1.012 (1.003,1.021) |
| Early Term | Land Cover | >66% | 1.040 (1.027,1.053) | 1.051 (1.032,1.069) | 1.059 (1.031,1.087) | 1.027 (1.016,1.037) |

**Table S2:** Pooled odds ratios and 95% confidence intervals for all outcomes, strata, and heat wave definitions. The consecutive-day heat wave definitions, are dichotomous exposure categories. The per °C results represent the odds ratio associated with a 1°C increase in the 4-day average degrees over the 97.5th percentile.

Table S3: State-specific results, stratified by maternal age

| State | Outcome | Level | 2+ consecutive days | 3+ consecutive days | 4 consecutive days | per °C |
|---|---|---|---|---|---|---|
| California | Preterm | <25 | 0.982 (0.959,1.004) | 1.005 (0.973,1.038) | 1.017 (0.969,1.068) | 1.015 (0.996,1.035) |
| California | Preterm | 25-34 | 1.014 (0.994,1.034) | 1.010 (0.982,1.038) | 1.019 (0.977,1.063) | 1.004 (0.988,1.021) |
| California | Preterm | 35+ | 1.026 (0.994,1.059) | 1.050 (1.003,1.099) | 1.091 (1.018,1.170) | 1.035 (1.009,1.063) |
| Florida | Preterm | <25 | 0.978 (0.927,1.033) | 0.964 (0.894,1.040) | 1.002 (0.899,1.116) | 0.983 (0.878,1.100) |
| Florida | Preterm | 25-34 | 1.003 (0.960,1.048) | 0.982 (0.923,1.045) | 1.013 (0.927,1.106) | 0.996 (0.904,1.098) |
| Florida | Preterm | 35+ | 1.006 (0.932,1.085) | 1.046 (0.942,1.162) | 1.035 (0.888,1.208) | 1.083 (0.914,1.283) |
| Georgia | Preterm | <25 | 0.988 (0.941,1.037) | 1.005 (0.942,1.073) | 0.932 (0.849,1.024) | 0.979 (0.920,1.042) |
| Georgia | Preterm | 25-34 | 0.981 (0.938,1.027) | 0.978 (0.920,1.039) | 1.046 (0.959,1.141) | 1.006 (0.948,1.067) |
| Georgia | Preterm | 35+ | 1.090 (1.005,1.182) | 1.100 (0.987,1.226) | 1.093 (0.935,1.278) | 1.096 (0.986,1.218) |
| Kansas | Preterm | <25 | 1.076 (0.981,1.181) | 1.043 (0.922,1.180) | 0.977 (0.823,1.159) | 1.042 (0.952,1.142) |
| Kansas | Preterm | 25-34 | 0.953 (0.876,1.038) | 0.960 (0.856,1.076) | 0.885 (0.754,1.039) | 0.925 (0.849,1.009) |
| Kansas | Preterm | 35+ | 0.996 (0.833,1.191) | 1.045 (0.826,1.321) | 0.937 (0.667,1.317) | 0.942 (0.788,1.127) |
| Nevada | Preterm | <25 | 0.950 (0.865,1.043) | 0.961 (0.853,1.082) | 0.929 (0.789,1.093) | 0.948 (0.876,1.026) |
| Nevada | Preterm | 25-34 | 0.978 (0.904,1.059) | 0.932 (0.842,1.032) | 0.911 (0.792,1.048) | 0.959 (0.899,1.023) |
| Nevada | Preterm | 35+ | 1.016 (0.890,1.160) | 1.006 (0.850,1.192) | 1.156 (0.926,1.444) | 1.043 (0.940,1.157) |
| New Jersey | Preterm | <25 | 1.033 (0.966,1.104) | 1.040 (0.945,1.145) | 1.127 (0.975,1.302) | 1.013 (0.954,1.074) |
| New Jersey | Preterm | 25-34 | 1.035 (0.988,1.084) | 1.020 (0.955,1.090) | 1.048 (0.946,1.162) | 1.007 (0.965,1.051) |
| New Jersey | Preterm | 35+ | 1.045 (0.971,1.124) | 1.057 (0.952,1.174) | 1.060 (0.895,1.257) | 1.054 (0.986,1.127) |
| North Carolina | Preterm | <25 | 1.032 (0.967,1.102) | 1.057 (0.969,1.153) | 1.026 (0.902,1.167) | 1.024 (0.952,1.102) |
| North Carolina | Preterm | 25-34 | 0.986 (0.930,1.045) | 0.990 (0.914,1.071) | 1.030 (0.920,1.153) | 1.026 (0.960,1.096) |
| North Carolina | Preterm | 35+ | 0.899 (0.805,1.005) | 0.948 (0.817,1.100) | 0.970 (0.780,1.205) | 0.950 (0.836,1.081) |
| Oregon | Preterm | <25 | 1.104 (1.009,1.207) | 1.164 (1.018,1.330) | 1.122 (0.897,1.402) | 1.074 (1.006,1.146) |
| Oregon | Preterm | 25-34 | 1.048 (0.970,1.132) | 1.087 (0.969,1.219) | 1.049 (0.866,1.270) | 1.038 (0.983,1.097) |
| Oregon | Preterm | 35+ | 1.104 (0.954,1.277) | 1.190 (0.958,1.478) | 1.302 (0.919,1.845) | 1.105 (1.002,1.218) |

Table S3: State-specific results, stratified by maternal age

| State | Outcome | Level | 2+ consecutive days | 3+ consecutive days | 4 consecutive days | per °C |
|---|---|---|---|---|---|---|
| California | Early Term | <25 | 1.033 (1.018,1.047) | 1.049 (1.029,1.070) | 1.037 (1.007,1.068) | 1.023 (1.012,1.035) |
| California | Early Term | 25-34 | 1.023 (1.012,1.034) | 1.019 (1.004,1.035) | 1.010 (0.987,1.033) | 1.014 (1.005,1.023) |
| California | Early Term | 35+ | 0.991 (0.973,1.010) | 1.005 (0.978,1.031) | 1.028 (0.987,1.070) | 1.009 (0.994,1.024) |
| Florida | Early Term | <25 | 0.988 (0.959,1.018) | 0.990 (0.950,1.033) | 0.986 (0.928,1.047) | 0.993 (0.932,1.058) |
| Florida | Early Term | 25-34 | 1.008 (0.985,1.031) | 1.033 (1.001,1.066) | 1.042 (0.996,1.091) | 1.044 (0.994,1.097) |
| Florida | Early Term | 35+ | 0.980 (0.939,1.024) | 0.998 (0.939,1.061) | 1.035 (0.945,1.133) | 1.007 (0.909,1.115) |
| Georgia | Early Term | <25 | 1.010 (0.981,1.039) | 1.038 (0.999,1.078) | 1.044 (0.989,1.102) | 1.004 (0.967,1.042) |
| Georgia | Early Term | 25-34 | 1.009 (0.985,1.034) | 0.990 (0.958,1.023) | 1.016 (0.970,1.064) | 0.994 (0.963,1.026) |
| Georgia | Early Term | 35+ | 0.995 (0.947,1.045) | 0.966 (0.904,1.033) | 0.955 (0.867,1.053) | 0.953 (0.894,1.015) |
| Kansas | Early Term | <25 | 0.992 (0.941,1.046) | 1.006 (0.937,1.081) | 1.049 (0.953,1.155) | 0.996 (0.945,1.050) |
| Kansas | Early Term | 25-34 | 0.992 (0.949,1.037) | 0.986 (0.929,1.046) | 0.951 (0.876,1.032) | 1.000 (0.958,1.044) |
| Kansas | Early Term | 35+ | 0.992 (0.889,1.107) | 0.938 (0.810,1.086) | 0.992 (0.811,1.213) | 1.048 (0.944,1.163) |
| Nevada | Early Term | <25 | 1.002 (0.952,1.056) | 0.996 (0.933,1.064) | 0.953 (0.870,1.044) | 0.990 (0.948,1.035) |
| Nevada | Early Term | 25-34 | 1.048 (1.006,1.093) | 1.045 (0.992,1.101) | 1.022 (0.952,1.098) | 1.012 (0.979,1.046) |
| Nevada | Early Term | 35+ | 0.971 (0.895,1.053) | 0.955 (0.860,1.059) | 0.941 (0.818,1.084) | 0.985 (0.924,1.050) |
| New Jersey | Early Term | <25 | 1.049 (1.008,1.092) | 1.077 (1.018,1.140) | 1.086 (0.997,1.183) | 1.035 (0.999,1.071) |
| New Jersey | Early Term | 25-34 | 0.994 (0.970,1.019) | 1.006 (0.970,1.042) | 1.033 (0.978,1.092) | 1.014 (0.992,1.037) |
| New Jersey | Early Term | 35+ | 0.961 (0.922,1.002) | 0.974 (0.916,1.035) | 0.938 (0.850,1.036) | 0.977 (0.939,1.017) |
| North Carolina | Early Term | <25 | 0.950 (0.913,0.988) | 0.933 (0.884,0.984) | 0.897 (0.829,0.971) | 0.941 (0.899,0.986) |
| North Carolina | Early Term | 25-34 | 0.997 (0.965,1.030) | 0.997 (0.954,1.041) | 1.000 (0.938,1.066) | 0.982 (0.946,1.019) |
| North Carolina | Early Term | 35+ | 0.994 (0.929,1.064) | 0.971 (0.886,1.064) | 1.034 (0.906,1.181) | 0.955 (0.883,1.033) |
| Oregon | Early Term | <25 | 1.024 (0.973,1.078) | 1.024 (0.949,1.105) | 1.020 (0.899,1.158) | 0.986 (0.948,1.025) |
| Oregon | Early Term | 25-34 | 1.009 (0.969,1.050) | 1.008 (0.951,1.069) | 0.965 (0.875,1.065) | 1.000 (0.972,1.029) |
| Oregon | Early Term | 35+ | 1.025 (0.948,1.109) | 1.053 (0.940,1.179) | 1.060 (0.876,1.283) | 1.000 (0.946,1.057) |

**Table S3:** State-specific odds ratios and 95% confidence intervals, stratified by maternal age. The consecutive-day heat wave definitions are dichotomous exposure categories. The per °C results represent the odds ratio associated with a 1°C increase in the 4-day average degrees over the 97.5th percentile.

Table S4: State-specific results, stratified by maternal education

| State | Outcome | Level | 2+ consecutive days | 3+ consecutive days | 4 consecutive days | per °C |
|---|---|---|---|---|---|---|
| California | Preterm | <HS | 1.003 (0.980,1.028) | 1.020 (0.986,1.055) | 1.032 (0.982,1.085) | 1.019 (0.999,1.039) |
| California | Preterm | HS/GED | 0.985 (0.960,1.010) | 0.988 (0.953,1.024) | 1.005 (0.951,1.062) | 1.002 (0.980,1.024) |
| California | Preterm | >HS | 1.020 (0.998,1.043) | 1.025 (0.993,1.058) | 1.046 (0.996,1.098) | 1.015 (0.996,1.033) |
| Florida | Preterm | <HS | 0.970 (0.901,1.043) | 0.949 (0.856,1.053) | 0.968 (0.834,1.124) | 0.982 (0.840,1.148) |
| Florida | Preterm | HS/GED | 0.951 (0.902,1.004) | 0.964 (0.894,1.039) | 0.958 (0.860,1.067) | 0.925 (0.822,1.040) |
| Florida | Preterm | >HS | 1.036 (0.991,1.084) | 1.028 (0.966,1.095) | 1.084 (0.991,1.186) | 1.093 (0.991,1.205) |
| Georgia | Preterm | <HS | 1.028 (0.963,1.097) | 1.096 (1.005,1.194) | 0.945 (0.836,1.069) | 1.031 (0.954,1.115) |
| Georgia | Preterm | HS/GED | 0.974 (0.922,1.028) | 0.961 (0.893,1.035) | 0.928 (0.834,1.032) | 0.935 (0.870,1.005) |
| Georgia | Preterm | >HS | 0.996 (0.951,1.044) | 0.986 (0.926,1.051) | 1.085 (0.991,1.188) | 1.032 (0.970,1.097) |
| Kansas | Preterm | <HS | 1.057 (0.925,1.207) | 1.007 (0.839,1.208) | 0.956 (0.738,1.238) | 0.951 (0.826,1.096) |
| Kansas | Preterm | HS/GED | 1.014 (0.909,1.132) | 0.937 (0.810,1.083) | 0.867 (0.707,1.063) | 0.944 (0.844,1.055) |
| Kansas | Preterm | >HS | 0.982 (0.903,1.067) | 1.045 (0.936,1.167) | 0.972 (0.833,1.133) | 0.999 (0.921,1.083) |
| Nevada | Preterm | <HS | 1.013 (0.912,1.125) | 1.082 (0.948,1.235) | 1.095 (0.917,1.308) | 1.012 (0.928,1.102) |
| Nevada | Preterm | HS/GED | 0.919 (0.834,1.014) | 0.828 (0.727,0.942) | 0.755 (0.627,0.910) | 0.894 (0.819,0.975) |
| Nevada | Preterm | >HS | 1.005 (0.920,1.099) | 0.970 (0.866,1.086) | 1.023 (0.880,1.190) | 0.988 (0.922,1.059) |
| New Jersey | Preterm | <HS | 1.046 (0.963,1.136) | 1.059 (0.941,1.192) | 1.122 (0.936,1.345) | 1.028 (0.954,1.107) |
| New Jersey | Preterm | HS/GED | 1.016 (0.957,1.079) | 0.968 (0.888,1.055) | 1.007 (0.884,1.148) | 0.975 (0.923,1.029) |
| New Jersey | Preterm | >HS | 1.040 (0.993,1.090) | 1.064 (0.995,1.138) | 1.073 (0.965,1.194) | 1.038 (0.994,1.083) |
| Oregon | Preterm | <HS | 1.123 (0.999,1.263) | 1.169 (0.981,1.392) | 1.048 (0.774,1.420) | 1.047 (0.964,1.137) |
| Oregon | Preterm | HS/GED | 1.111 (1.007,1.225) | 1.199 (1.036,1.389) | 1.117 (0.876,1.423) | 1.087 (1.011,1.169) |
| Oregon | Preterm | >HS | 1.040 (0.960,1.126) | 1.082 (0.961,1.219) | 1.212 (1.000,1.469) | 1.063 (1.006,1.124) |
| California | Early Term | <HS | 1.025 (1.010,1.040) | 1.050 (1.029,1.072) | 1.036 (1.005,1.069) | 1.021 (1.009,1.033) |
| California | Early Term | HS/GED | 1.026 (1.011,1.041) | 1.029 (1.008,1.051) | 1.028 (0.995,1.061) | 1.021 (1.008,1.033) |
| California | Early Term | >HS | 1.014 (1.002,1.026) | 1.009 (0.992,1.026) | 1.016 (0.990,1.042) | 1.011 (1.001,1.020) |
| Florida | Early Term | <HS | 0.979 (0.938,1.023) | 0.974 (0.917,1.035) | 0.972 (0.891,1.061) | 0.970 (0.883,1.065) |
| Florida | Early Term | HS/GED | 0.989 (0.961,1.019) | 1.017 (0.976,1.060) | 1.059 (0.997,1.125) | 1.036 (0.972,1.105) |
| Florida | Early Term | >HS | 1.007 (0.984,1.030) | 1.016 (0.984,1.049) | 1.012 (0.966,1.061) | 1.016 (0.966,1.068) |
| Georgia | Early Term | <HS | 1.025 (0.985,1.067) | 1.034 (0.980,1.091) | 0.986 (0.914,1.063) | 0.974 (0.925,1.025) |
| Georgia | Early Term | HS/GED | 1.001 (0.970,1.033) | 1.006 (0.965,1.050) | 1.046 (0.986,1.111) | 1.015 (0.974,1.058) |
| Georgia | Early Term | >HS | 1.012 (0.986,1.037) | 0.993 (0.960,1.027) | 1.014 (0.967,1.065) | 0.998 (0.966,1.031) |
| Kansas | Early Term | <HS | 1.025 (0.946,1.111) | 0.921 (0.824,1.031) | 0.959 (0.820,1.122) | 0.978 (0.897,1.066) |
| Kansas | Early Term | HS/GED | 1.029 (0.967,1.095) | 1.023 (0.940,1.113) | 1.037 (0.924,1.162) | 1.018 (0.956,1.085) |
| Kansas | Early Term | >HS | 0.975 (0.934,1.018) | 0.991 (0.936,1.050) | 0.989 (0.915,1.069) | 1.009 (0.968,1.051) |
| Nevada | Early Term | <HS | 0.983 (0.924,1.046) | 0.977 (0.903,1.058) | 0.915 (0.819,1.023) | 0.989 (0.937,1.043) |
| Nevada | Early Term | HS/GED | 1.071 (1.016,1.129) | 1.063 (0.994,1.137) | 1.017 (0.927,1.115) | 1.033 (0.989,1.080) |
| Nevada | Early Term | >HS | 1.012 (0.966,1.059) | 1.002 (0.946,1.063) | 0.996 (0.921,1.078) | 0.986 (0.951,1.023) |
| New Jersey | Early Term | <HS | 0.984 (0.934,1.036) | 0.987 (0.917,1.063) | 0.968 (0.864,1.084) | 1.011 (0.966,1.057) |
| New Jersey | Early Term | HS/GED | 1.038 (1.003,1.075) | 1.084 (1.031,1.139) | 1.136 (1.053,1.226) | 1.053 (1.021,1.086) |
| New Jersey | Early Term | >HS | 0.988 (0.964,1.013) | 0.991 (0.957,1.027) | 0.993 (0.939,1.050) | 0.991 (0.968,1.013) |
| Oregon | Early Term | <HS | 1.017 (0.950,1.089) | 1.020 (0.922,1.128) | 1.100 (0.937,1.292) | 0.985 (0.937,1.037) |
| Oregon | Early Term | HS/GED | 0.988 (0.936,1.043) | 0.939 (0.866,1.018) | 0.975 (0.852,1.116) | 0.977 (0.938,1.019) |
| Oregon | Early Term | >HS | 1.016 (0.975,1.058) | 1.041 (0.981,1.105) | 0.926 (0.836,1.025) | 1.004 (0.975,1.034) |

**Table S4:** State-specific odds ratios and 95% confidence intervals, stratified by maternal education. The consecutive-day heat wave definitions are dichotomous exposure categories. The per °C results represent the odds ratio associated with a 1°C increase in the 4-day average degrees over the 97.5th percentile.

Table S5: State-specific results, stratified by land cover

| State | Outcome | Level | 2+ consecutive days | 3+ consecutive days | 4 consecutive days | per °C |
|---|---|---|---|---|---|---|
| California | Preterm | <25% | 1.016 (0.994,1.039) | 1.007 (0.976,1.039) | 0.987 (0.942,1.034) | 1.007 (0.989,1.025) |
| California | Preterm | 25-66% | 1.030 (1.007,1.055) | 1.050 (1.015,1.086) | 1.090 (1.034,1.149) | 1.027 (1.007,1.047) |
| California | Preterm | >66% | 0.990 (0.967,1.014) | 1.005 (0.972,1.039) | 1.015 (0.964,1.068) | 1.008 (0.987,1.029) |
| Florida | Preterm | <25% | 1.017 (0.982,1.053) | 1.022 (0.974,1.072) | 1.063 (0.993,1.137) | 1.057 (0.984,1.136) |
| Florida | Preterm | 25-66% | 0.949 (0.889,1.013) | 0.936 (0.848,1.033) | 0.873 (0.748,1.020) | 0.871 (0.730,1.038) |
| Florida | Preterm | >66% | 0.825 (0.525,1.297) | 0.657 (0.330,1.307) | 0.519 (0.151,1.785) | 0.562 (0.170,1.858) |
| Georgia | Preterm | <25% | 1.000 (0.968,1.032) | 1.007 (0.965,1.051) | 1.013 (0.953,1.076) | 1.016 (0.974,1.059) |
| Georgia | Preterm | 25-66% | 0.977 (0.884,1.080) | 0.966 (0.843,1.106) | 0.911 (0.747,1.111) | 0.911 (0.806,1.030) |
| Georgia | Preterm | >66% | 1.027 (0.523,2.016) | 1.640 (0.669,4.019) | 1.962 (0.584,6.591) | 0.756 (0.249,2.298) |
| Kansas | Preterm | <25% | 1.006 (0.945,1.070) | 0.995 (0.915,1.080) | 0.941 (0.839,1.056) | 0.978 (0.920,1.040) |
| Kansas | Preterm | 25-66% | 0.963 (0.817,1.137) | 0.970 (0.780,1.207) | 0.764 (0.552,1.058) | 0.870 (0.727,1.041) |
| Kansas | Preterm | >66% | 0.843 (0.231,3.076) | 1.432 (0.304,6.742) | 0.763 (0.070,8.359) | 1.069 (0.346,3.300) |
| Nevada | Preterm | <25% | 0.960 (0.867,1.063) | 0.922 (0.808,1.053) | 0.941 (0.788,1.123) | 0.943 (0.868,1.023) |
| Nevada | Preterm | 25-66% | 0.987 (0.905,1.075) | 0.961 (0.860,1.073) | 0.991 (0.855,1.149) | 0.984 (0.920,1.053) |
| Nevada | Preterm | >66% | 0.966 (0.879,1.063) | 0.979 (0.867,1.105) | 0.925 (0.778,1.100) | 0.970 (0.890,1.057) |
| New Jersey | Preterm | <25% | 1.000 (0.953,1.050) | 0.997 (0.931,1.069) | 1.037 (0.930,1.155) | 1.011 (0.966,1.058) |
| New Jersey | Preterm | 25-66% | 1.079 (1.013,1.150) | 1.044 (0.953,1.143) | 1.036 (0.900,1.193) | 1.038 (0.981,1.097) |
| New Jersey | Preterm | >66% | 1.030 (0.963,1.102) | 1.050 (0.953,1.157) | 1.084 (0.935,1.258) | 0.989 (0.932,1.050) |
| North Carolina | Preterm | <25% | 0.983 (0.944,1.024) | 1.001 (0.948,1.057) | 1.010 (0.933,1.094) | 1.011 (0.965,1.059) |
| North Carolina | Preterm | 25-66% | 1.095 (0.892,1.344) | 1.150 (0.877,1.507) | 1.306 (0.900,1.895) | 1.124 (0.917,1.377) |
| North Carolina | Preterm | >66% | 0.444 (0.085,2.308) | 0.493 (0.079,3.080) | 1.449 (0.221,9.510) | 0.660 (0.159,2.738) |
| Oregon | Preterm | <25% | 1.091 (1.022,1.164) | 1.172 (1.064,1.291) | 1.079 (0.920,1.265) | 1.073 (1.023,1.126) |
| Oregon | Preterm | 25-66% | 0.998 (0.903,1.103) | 1.058 (0.912,1.227) | 1.158 (0.901,1.488) | 1.029 (0.961,1.102) |
| Oregon | Preterm | >66% | 1.230 (0.939,1.611) | 0.989 (0.644,1.518) | 1.886 (0.976,3.645) | 1.171 (0.986,1.391) |

Table S5: State-specific results, stratified by land cover

| State | Outcome | Level | 2+ consecutive days | 3+ consecutive days | 4 consecutive days | per °C |
|---|---|---|---|---|---|---|
| California | Early Term | <25% | 1.015 (1.002,1.028) | 1.009 (0.991,1.027) | 0.990 (0.964,1.017) | 1.007 (0.997,1.017) |
| California | Early Term | 25-66% | 1.017 (1.003,1.030) | 1.038 (1.018,1.058) | 1.035 (1.005,1.067) | 1.020 (1.009,1.030) |
| California | Early Term | >66% | 1.041 (1.027,1.056) | 1.051 (1.031,1.072) | 1.068 (1.038,1.100) | 1.027 (1.016,1.039) |
| Florida | Early Term | <25% | 0.992 (0.974,1.010) | 1.015 (0.990,1.041) | 1.031 (0.995,1.069) | 1.022 (0.984,1.062) |
| Florida | Early Term | 25-66% | 1.010 (0.976,1.046) | 0.990 (0.939,1.044) | 0.954 (0.877,1.038) | 0.948 (0.860,1.044) |
| Florida | Early Term | >66% | 1.108 (0.901,1.364) | 0.998 (0.726,1.372) | 0.922 (0.536,1.586) | 0.884 (0.481,1.627) |
| Georgia | Early Term | <25% | 1.013 (0.995,1.031) | 1.012 (0.988,1.036) | 1.028 (0.994,1.063) | 1.004 (0.981,1.028) |
| Georgia | Early Term | 25-66% | 0.999 (0.942,1.059) | 1.013 (0.937,1.095) | 1.032 (0.923,1.154) | 0.976 (0.912,1.046) |
| Georgia | Early Term | >66% | 0.658 (0.426,1.017) | 0.877 (0.517,1.487) | 1.378 (0.698,2.721) | 0.763 (0.468,1.243) |
| Kansas | Early Term | <25% | 0.993 (0.961,1.026) | 0.987 (0.945,1.032) | 0.969 (0.912,1.030) | 0.989 (0.957,1.021) |
| Kansas | Early Term | 25-66% | 0.978 (0.891,1.074) | 0.953 (0.841,1.080) | 1.030 (0.871,1.218) | 1.066 (0.974,1.166) |
| Kansas | Early Term | >66% | 0.948 (0.516,1.740) | 0.946 (0.430,2.078) | 1.328 (0.433,4.075) | 1.007 (0.530,1.913) |
| Nevada | Early Term | <25% | 1.017 (0.966,1.072) | 1.054 (0.987,1.126) | 0.968 (0.884,1.059) | 0.997 (0.957,1.038) |
| Nevada | Early Term | 25-66% | 1.005 (0.958,1.054) | 0.971 (0.913,1.031) | 0.976 (0.899,1.059) | 0.984 (0.948,1.021) |
| Nevada | Early Term | >66% | 1.048 (0.993,1.106) | 1.024 (0.957,1.097) | 0.981 (0.891,1.080) | 1.013 (0.965,1.064) |
| New Jersey | Early Term | <25% | 0.986 (0.961,1.011) | 0.989 (0.954,1.026) | 1.017 (0.961,1.076) | 1.003 (0.979,1.027) |
| New Jersey | Early Term | 25-66% | 1.007 (0.972,1.044) | 1.015 (0.964,1.068) | 1.014 (0.937,1.098) | 0.999 (0.968,1.031) |
| New Jersey | Early Term | >66% | 1.018 (0.979,1.058) | 1.057 (0.999,1.118) | 1.037 (0.949,1.132) | 1.026 (0.993,1.060) |
| North Carolina | Early Term | <25% | 0.978 (0.956,1.001) | 0.976 (0.946,1.007) | 0.976 (0.932,1.022) | 0.971 (0.945,0.997) |
| North Carolina | Early Term | 25-66% | 1.020 (0.892,1.166) | 0.964 (0.809,1.149) | 0.826 (0.636,1.074) | 0.961 (0.830,1.113) |
| North Carolina | Early Term | >66% | 0.931 (0.342,2.535) | 0.638 (0.124,3.297) | 0.523 (0.057,4.817) | 0.952 (0.221,4.089) |
| Oregon | Early Term | <25% | 1.025 (0.990,1.060) | 1.013 (0.963,1.066) | 0.990 (0.910,1.076) | 0.992 (0.966,1.018) |
| Oregon | Early Term | 25-66% | 0.961 (0.911,1.014) | 0.979 (0.905,1.060) | 0.950 (0.829,1.090) | 0.978 (0.942,1.015) |
| Oregon | Early Term | >66% | 1.129 (0.978,1.303) | 1.188 (0.972,1.453) | 1.116 (0.775,1.608) | 1.059 (0.969,1.158) |

**Table S5:** State-specific odds ratios and 95% confidence intervals, stratified by land cover. The consecutive-day heat wave definitions are dichotomous exposure categories. The per °C results represent the odds ratio associated with a 1°C increase in the 4-day average degrees over the 97.5th percentile.

Table S6: State-specific results, stratified by social deprivation index

| State | Outcome | Level | 2+ consecutive days | 3+ consecutive days | 4 consecutive days | per °C |
|---|---|---|---|---|---|---|
| California | Preterm | Low | 1.011 (0.989,1.034) | 1.020 (0.987,1.054) | 1.021 (0.969,1.075) | 1.009 (0.991,1.029) |
| California | Preterm | High | 1.013 (0.996,1.030) | 1.019 (0.996,1.043) | 1.028 (0.993,1.064) | 1.016 (1.002,1.030) |
| Florida | Preterm | Low | 1.005 (0.966,1.045) | 1.013 (0.960,1.070) | 1.034 (0.957,1.117) | 1.061 (0.975,1.154) |
| Florida | Preterm | High | 0.994 (0.946,1.044) | 0.985 (0.919,1.057) | 1.013 (0.915,1.123) | 0.969 (0.868,1.081) |
| Georgia | Preterm | Low | 1.005 (0.964,1.048) | 0.995 (0.941,1.053) | 1.047 (0.967,1.135) | 1.005 (0.952,1.060) |
| Georgia | Preterm | High | 0.990 (0.947,1.034) | 1.014 (0.956,1.076) | 0.959 (0.881,1.044) | 1.002 (0.947,1.061) |
| Kansas | Preterm | Low | 1.016 (0.950,1.087) | 1.019 (0.931,1.115) | 0.972 (0.858,1.101) | 0.989 (0.926,1.057) |
| Kansas | Preterm | High | 0.957 (0.854,1.071) | 0.921 (0.792,1.072) | 0.777 (0.625,0.966) | 0.898 (0.797,1.012) |
| Nevada | Preterm | Low | 0.996 (0.920,1.078) | 0.961 (0.867,1.065) | 0.992 (0.865,1.137) | 0.974 (0.915,1.037) |
| Nevada | Preterm | High | 0.952 (0.885,1.026) | 0.950 (0.865,1.045) | 0.925 (0.811,1.055) | 0.960 (0.901,1.023) |
| New Jersey | Preterm | Low | 1.033 (0.987,1.081) | 1.018 (0.954,1.087) | 1.025 (0.924,1.136) | 1.018 (0.975,1.062) |
| New Jersey | Preterm | High | 1.024 (0.975,1.076) | 1.028 (0.958,1.103) | 1.074 (0.965,1.195) | 1.008 (0.965,1.052) |
| North Carolina | Preterm | Low | 0.963 (0.913,1.015) | 0.966 (0.899,1.038) | 0.941 (0.847,1.046) | 0.974 (0.916,1.036) |
| North Carolina | Preterm | High | 1.018 (0.959,1.082) | 1.060 (0.978,1.150) | 1.131 (1.008,1.269) | 1.069 (0.999,1.143) |
| Oregon | Preterm | Low | 1.072 (1.006,1.143) | 1.135 (1.032,1.248) | 1.114 (0.950,1.306) | 1.066 (1.019,1.117) |
| Oregon | Preterm | High | 1.057 (0.959,1.165) | 1.121 (0.969,1.296) | 1.140 (0.901,1.442) | 1.056 (0.987,1.130) |
| California | Early Term | Low | 1.012 (1.000,1.024) | 1.020 (1.002,1.038) | 1.026 (0.998,1.054) | 1.011 (1.001,1.020) |
| California | Early Term | High | 1.031 (1.021,1.041) | 1.038 (1.024,1.052) | 1.029 (1.008,1.051) | 1.021 (1.013,1.030) |
| Florida | Early Term | Low | 1.005 (0.985,1.025) | 1.025 (0.997,1.054) | 1.030 (0.989,1.073) | 1.021 (0.978,1.067) |
| Florida | Early Term | High | 0.982 (0.955,1.008) | 0.982 (0.945,1.021) | 0.994 (0.938,1.053) | 0.992 (0.933,1.055) |
| Georgia | Early Term | Low | 1.014 (0.992,1.037) | 1.000 (0.970,1.030) | 1.023 (0.980,1.067) | 1.004 (0.976,1.034) |
| Georgia | Early Term | High | 1.007 (0.981,1.033) | 1.028 (0.994,1.064) | 1.037 (0.988,1.089) | 0.996 (0.963,1.031) |
| Kansas | Early Term | Low | 0.990 (0.955,1.025) | 0.991 (0.945,1.040) | 0.988 (0.926,1.053) | 0.997 (0.964,1.032) |
| Kansas | Early Term | High | 1.001 (0.939,1.067) | 0.961 (0.882,1.048) | 0.940 (0.833,1.061) | 0.994 (0.931,1.062) |
| Nevada | Early Term | Low | 1.005 (0.964,1.047) | 1.004 (0.953,1.058) | 0.969 (0.904,1.040) | 0.986 (0.955,1.018) |
| Nevada | Early Term | High | 1.040 (0.997,1.085) | 1.021 (0.968,1.078) | 0.980 (0.908,1.056) | 1.007 (0.971,1.045) |
| New Jersey | Early Term | Low | 0.990 (0.967,1.014) | 0.990 (0.957,1.024) | 1.008 (0.955,1.063) | 0.995 (0.973,1.017) |
| New Jersey | Early Term | High | 1.010 (0.982,1.040) | 1.039 (0.997,1.083) | 1.039 (0.975,1.107) | 1.024 (0.999,1.050) |
| North Carolina | Early Term | Low | 0.953 (0.925,0.982) | 0.961 (0.923,1.000) | 0.965 (0.910,1.023) | 0.950 (0.918,0.985) |
| North Carolina | Early Term | High | 1.018 (0.983,1.054) | 0.998 (0.951,1.046) | 0.979 (0.913,1.050) | 0.997 (0.958,1.037) |
| Oregon | Early Term | Low | 1.010 (0.976,1.044) | 0.987 (0.939,1.038) | 0.964 (0.887,1.048) | 0.981 (0.957,1.006) |
| Oregon | Early Term | High | 1.010 (0.959,1.063) | 1.064 (0.986,1.148) | 1.027 (0.903,1.169) | 1.012 (0.974,1.051) |

Table S6: State-specific odds ratios and 95% confidence intervals, stratified by social deprivation index. The consecutive-day heat wave definitions are dichotomous exposure categories. The per °C results represent the odds ratio associated with a 1°C increase in the 4-day average degrees over the 97.5th percentile.
